# Dimensions of Autistic Traits Rated by Parents of Children and Adolescents with Suspected Autism Spectrum Disorders

**DOI:** 10.1007/s10803-020-04850-4

**Published:** 2021-01-08

**Authors:** Teresa Del Giudice, Christina Dose, Anja Görtz-Dorten, Jennifer Steiner, Nicole Bruning, Hannah Bell, Pamela Roland, Daniel Walter, Michaela Junghänel, Manfred Döpfner

**Affiliations:** 1 Department of Child and Adolescent Psychiatry, Psychosomatics and Psychotherapy, Faculty of Medicine and University Hospital Cologne, University of Cologne, Cologne, Germany; 2 School of Child and Adolescent Cognitive Behavior Therapy (AKiP), Faculty of Medicine and University Hospital Cologne, University of Cologne, Pohligstraße 9, 50969 Cologne, Germany

**Keywords:** Autism spectrum disorders, Children and adolescents, Parent ratings, Factor structure, Reliability, Validity

## Abstract

To examine the factor structure of autism spectrum disorder (ASD) and the psychometric properties of the German Symptom Checklist for Autism Spectrum Disorders (SCL-ASD). Data were collected from 312 clinical referrals with suspected ASD (2–18 years). Confirmatory factor analyses and analyses of reliability, convergent and divergent validity were performed. A bifactor model with one general ASD factor and two specific factors (interaction-communication; restricted, repetitive behaviors) provided an adequate data fit. Internal consistencies of the SCL-ASD subscales and the total scale were > .70. Correlations with measures of ASD traits were higher than correlations with measures of externalizing and internalizing symptoms. The results support a factor structure consistent with DSM-5/ICD-11 criteria. The SCL-ASD has sound psychometric properties.

**Supplementary information:**

The online version of this article (10.1007/s10803-020-04850-4) contains supplementary material, which is available to authorized users.

## Introductions

Despite many research efforts over the past years focusing on the classification of autism spectrum disorders (ASD), essential questions about their underlying structure remain unresolved (Happé 2011; Kim et al. 2018; Lecavalier et al. 2009). The current fifth edition of the Diagnostic and Statistical Manual of Mental Disorders (DSM-5; American Psychiatric Association 2013) and the latest edition of the International Classification of Diseases (ICD-11; World Health Organization 2018) conceptualize the autism syndrome as a two-domain construct of interaction and social-communication deficits (INT-CO) on the one hand and restricted and repetitive interests/behaviors (RRB) on the other. This marks a substantial change from the long-prevailing fourth edition of the DSM (DSM-IV; American Psychiatric Association 1994) and tenth edition of the ICD (ICD-10; World Health Organization 1992), which classified autism as a triad of observable impairments in social interaction (INT), deficits in communication (CO) and RRB. This modification reflects the results of factor-analytic studies which only provided equivocal support for the factor structure derived from the DSM-IV/ICD-10 criteria. Although some studies indicated that a correlated-facto^2012^rs model with three factors according to the DSM-IV/ICD-10 criteria was the most suitable to reflect the structure of ratings of autistic traits (e.g. Beuker et al. 2012; Lecavalier et al. 2009; Sipes and Matson 2014), others showed that a two-domain conceptualization of autism provided the best fit to the data (this latter solution is similar to the DSM-5 model; e.g. Frazier et al. 2014,2008; Gotham et al. 2008,2007; Guthrie et al. 2013; Moulton et al. 2019; Snow et al. 2009). Moreover, some authors proposed models differing substantially from both the model postulated by the DSM-IV and that assumed by the DSM-5 (e.g. Bitsika and Sharpley 2018; Matson et al. 2009; Mirenda et al. 2010). The authors of a recently published study attempted to integrate competing models using Goldberg’s bass-ackwards method (Kim et al. 2018). They found a five-level hierarchy of factor models at various levels of resolution, with each level comprising a different factor solution (from a one-factor to a five-factor solution). In this framework, every level is a factor solution in itself; that is, the resulting structure does not imply subordinate or superordinate relationships as they are described in higher-order models. Instead, the different factor solutions organize ASD symptoms at different levels of resolution and the resulting overall structure indicates how the different solutions are interrelated. Notably, only the three- to five-factor solutions showed a good fit to the data, whereas the one-factor and the two-factor solutions only provided a poor to fair data fit.

More recently, studies examining bifactor models of ASD symptoms have provided an interesting impulse for research (Murray et al. 2017; Posserud et al. 2013). In contrast to correlated-factors models (like 2- or 3-correlated-factors solutions based on DSM-IV/ICD-10 or DSM-5/ICD-11 criteria, respectively), bifactor models test the presence of a general factor (g-factor) and further examine whether meaningful specific factors coexist alongside the g-factor (Chen et al. 2012,2006; Reise 2012). In a bifactor model, the general factor (here: “autism”) influences all items, whereas the specific factors (here: “INT, CO and RRB” or “INT/CO, RRB”) affect a specific subset of items. The general factor and the specific factors are uncorrelated and compete equally to explain variance. Thus, in bifactor models of autism, it is examined whether two or three specific dimensions (e.g. according to DSM and ICD) can be identified that exist beyond the general autism factor. Due to the descriptive nature of the classification systems, which basically provide an assignment of symptoms to domains on a first-order level, we consider them open to the deduction of different hypotheses on higher-order structures. Thus, we think that the bifactor approach described here is also consistent with the DSM and ICD classification of autistic traits.

To date, few studies have addressed the question of the existence of a general factor versus specific factors of autism. Snow et al. (2009) examined the fit of a bifactor model to data gathered with the *Autism Diagnostic Interview-Revised* (ADI-R; Lord et al. 1994) and were unable to detect a better fit compared to correlated models. Posserud et al. (2013) analyzed the psychometric properties of the *Autism Symptom Self-Report* for adolescents and adults (ASSERT) and found that a bifactor model with one general factor and two domain-specific factors (INT/CO, RRB) provided the best fit to the data. Murray et al. (2017) demonstrated in an adult sample that the items of the *Autism Spectrum Quotient* (AQ; Baron-Cohen et al. 2001) mainly reflected a general factor. They recommended the use of a bifactor measurement model when testing hypotheses on specific symptoms, as results on specific symptoms are biased by the influence of the general factor. For an overview of the three aforementioned studies, see also Table S1 in the supplementary material.

Another important issue considering the structure of autistic traits is the possibility of its change over time. Many previous studies considered a wide age range without adjusting for possible influences of age (e.g., 1–16 years, Gotham et al. 2008; 4–18 years, Snow et al. 2009; 2–47 years, Tadevosyan-Leyfer et al. 2003), others regarded only a small age range and, thus, do not allow for conclusions for other age groups (e.g., structure at 18 months, Beuker et al. 2012; 12–30 months; Guthrie et al. 2013). Frazier et al. demonstrated stability across age groups for both a two-factor model and a three-factor model of the ADI-R (Frazier et al. 2008) and measurement invariance of a two-factor model of the *Social Responsiveness Scale* (SRS; Constantino et al. 2003) across different age groups (Frazier et al. 2014). Duku et al. (2013) reported good overall fit of a second-order model of the ADI-R, which was consistent with DSM-5 criteria, but found that this model was not equivalent across different subgroups (divided by age, sex, and verbal ability). However, they demonstrated measurement invariance for a first-order six-factor model across their subgroups.

The present study examined the psychometric properties of a German questionnaire to assess ASD symptoms, the *Symptom Checklist for Autism Spectrum Disorders* (SCL-ASD; Döpfner et al. 2008), and uses this questionnaire to analyze the factor structure of ASD in a clinical sample of children with suspected ASD (2–18 years). The SCL-ASD is part of the German Diagnostic System for Mental Disorders in Childhood and Adolescence (DISYPS-II, Döpfner et al. 2008), which comprises caregiver-rated questionnaires, self-rated questionnaires and clinician-rated diagnostic checklists for the assessment of mental disorders in children and adolescents according to DSM-IV and ICD-10. Several instruments belonging to this diagnostic system are frequently used in Germany. However, the psychometric properties and the factor structure of the SCL-ASD have not been examined, yet. Regarding the factor structure of the SCL-ASD, we compared existing models that have been previously proposed and mostly independently validated (unidimensional model, 3-factor model, 2-factor model, bifactor model). In doing so, this is one of the few studies to include the examination of a bifactor model. Specifically, we examined whether a three-factor solution derived from DSM-IV/ICD-10 or a two-factor solution according to DSM-5/ICD-11 provides a better fit to the data, and whether the data are congruent with a hierarchical model that identifies a general factor of autism plus different domain-specific factors (INT/CO and RRB).

Due to the large age range in our sample and in order to take into account the possibility of different structures of ASD at different ages, we additionally examined the measurement invariance of the factor structure of the SCL-ASD across age groups.

Furthermore, we analyzed the reliability, the convergent validity and the divergent validity of the SCL-ASD. In this regard, we hypothesized that the subscales of the SCL-ASD would show higher correlations with the subscales of other instruments assessing ASD traits than with subscales assessing symptoms of other mental disorders.

## Methods

### Study Data

We used a clinical sample of 312 children and adolescents aged between 2 and 18 years (*M* = 10.5, *SD* = 3.7; 87% boys; see Table 1) who sought help at the School for Child and Adolescent Cognitive Behavior Therapy and the Department of Child and Adolescent Psychiatry, Psychosomatics and Psychotherapy at the University of Cologne, Germany, because of suspected ASD. Clinical diagnoses were made using a semi-structured, ICD-10- and DSM-IV-based clinical interview with the patients and their parents (Döpfner et al. 2008). Ninety percent of the children met ICD-10 criteria for ASD (47% Asperger’s syndrome; 41% infantile autism, 2% atypical autism). The remaining 10% did not meet the criteria for ASD but did meet the criteria for another ICD-10 diagnosis (primarily Attention-deficit/Hyperactivity disorder (ADHD)). We included both children with and without a formal diagnosis of ASD as autistic traits are common in the general population as well as in psychiatric patients, at least those with mood disorders (Constantino and Todd 2003; Pine et al. 2008), and as we aimed to increase the variance in the ratings of ASD symptoms.

**Table 1:** Sample characteristics by age groups

Age group	*n*	Gender (% boys)	% ASD (ICD-10)
Toddlers (2–4 years)	11	91	90
Early childhood (5–8 years)	89	92	94
Middle childhood (9–12 years)	116	85	93
Adolescents (13 + years)	96	83	82

Sample size *n* = 312

*ASD* autism spectrum disorder

### Measures

The *SCL-ASD parent rating* (Döpfner et al. 2008) is part of the German Diagnostic System for Mental Disorders in Childhood and Adolescence II (DISYPS-II) and measures symptoms of ASD according to DSM-IV and ICD-10. The parent-report questionnaire comprises 14 items that are rated with regard to their severity on a 4-point Likert-type scale ranging from 0 (“not at all”) to 3 (“very much”), with higher scores indicating higher symptom severity. Corresponding to DSM-IV and ICD-10 criteria, the items include questions which refer to deficits in communication (*CO*; items 1–6; e.g. “He/She shows marked impairment in the use of multiple nonverbal behaviors such as eye-to-eye gaze, facial expression, body postures, and gestures to regulate social interaction.”), impairments in social interaction (*INT*; items 7–10; e.g. “He/She shows a lack of varied, spontaneous make-believe play or social imitative play which is inappropriate for his/her developmental level.”) and restricted, repetitive interests/behaviors (RRB; items 11–14, e.g. “He/She shows apparently inflexible adherence to specific, nonfunctional routines or rituals.”). In this study, besides the 3-factor solution according to DSM-IV and ICD-10 (INT, CO, RRB), we examined a 2-factor solution according to DSM-5 and ICD-11 (INT-CO, RRB). For this purpose, items were aggregated to the subscales *INT-CO* (items 1–6, item 8, item 10) and *RRB* (item 9, items 11–14). Item 7 (“He/She shows a delay in, or total lack of, the development of spoken language.”) was deleted because the diagnostic criterion of delay in or complete lack of development of expressive language has been eliminated in the DSM-5. Moreover, also in line with the DSM-5, item 9 (“He/She shows stereotyped and repetitive use of language or idiosyncratic language.”) was shifted to the *RRB* subscale. The questionnaire has not yet been examined psychometrically.

The *Marburg Rating Scale for Asperger's Syndrome (*MBAS; Kamp-Becker et al. 2005) is a screening instrument for high-functioning autistic disorders for rating by caregivers of children, adolescents and young adults aged between 6 and 24 years. This scale consists of 65 questions, which address reciprocal social interaction, language and communication, and RRB (corresponding to DSM-IV and ICD-10 diagnostic criteria) on the following four scales: (1) *Theory of Mind, Social Contact and Play*, (2) *Joint Attention, Facial Expression, Gesture*, (3) *Stereotyped and Inadequate Behavior*, and (4) *Special Interests, Conspicuous Speech, Motor Activity*. Each item is rated on a 5-point Likert-type scale ranging from 0 (“never”) to 5 (“always”) referring to current symptoms and to symptoms that occurred between the age of 4 and 5 years. Notably, the original German version has shown satisfactory internal consistency (α = .91) and convergent validity with the ADI-R (*r* = .61; Kamp-Becker et al. 2005).

The *Autism Diagnostic Interview-Revised* (ADI-R; Lord et al. 1994) is a semi-structured, clinical interview conducted with caregivers of children and adults with suspected autism or autism spectrum disorders. The interview is composed of 93 items and asks about current behavior and behaviors that occurred during specific age periods. Corresponding to both DSM-IV and ICD-10, the questions focus on the following three domains: *CO*, *INT*, and *RRB*. Beyond that, the measure includes items relevant for treatment planning. The clinician scores all of the caregiver’s responses on a scale ranging from 0 to 7. Moreover, the clinician may note an “8” for “not applicable” or a “9” for “not known or asked”. A diagnosis of autism is made if scores in all three domains meet or exceed the specified cut-offs and if the onset of the disorder was evident by the age of 36 months. The subscales of the German version of the ADI-R demonstrated satisfactory internal consistency (α = .64 to α = .91; Bölte, Rühl, Schmötzer and Poustka 2006). Factor-analytic studies yielded equivocal results: Some studies showed that a three-domain conceptualization of the ADI-R provided the best fit to the data, while others proposed a two-factor solution (similar to the DSM-5 model) and others still suggested models that differed substantially from the DSM-IV and DSM-5 structures (for an overview see Shuster et al. 2014).

The *Child Behavior Checklist* (CBCL; Achenbach and Rescorla 2001) is a parent-rated questionnaire which assesses a broad spectrum of child behavioral and emotional problems. This checklist consists of 118 problem behavior items associated with two superordinate scales: the *Externalizing Problems* scale (including symptoms of conduct disorder and oppositional defiant disorder; e.g. “cruel to animals”) and the *Internalizing Problems* scale (including anxious and depressed symptoms; e.g. “complains of loneliness”; “too shy or timid”). Furthermore, the items can be aggregated to eight syndrome scales: *Aggressive Behavior*, *Anxious/Depressed*, *Attention Problems*, *Rule-Breaking Behavior*, *Somatic Complaints*, S*ocial Problems*, *Thought Problems*, and *Withdrawn/Depressed*. Each item is rated on a 3-point Likert-type scale ranging from 0 (“not true”) to 2 (“true”) referring to the child’s behavior in the past six months. Higher scores indicate higher symptom severity. The German version of the CBCL is a highly reliable rating scale (α = .69 to α = .93). Furthermore, all subscale scores and the total score have demonstrated factorial validity (Döpfner et al. 2014)

The Social Communication Questionnaire (SCQ; Rutter, Bailey and Lord 2003) is a parent-rated measure of ASD symptomatology and is suitable for children aged 4 years and older. This questionnaire is available in two versions, *Lifetime* and *Current*, each consisting of 40 binary items (yes/no), and each with a cut-off score of 15. Corresponding to the DSM-IV, the items include questions referring to reciprocal social interaction, language and communication, and repetitive, stereotyped patterns of behavior. Psychometric analysis yielded satisfactory internal consistency and validity of the questionnaire (Rutter et al. 2003). In the present study, the German adaptation of the SCQ (“*Fragebogen zur sozialen Kommunikation*”, FSK; Bölte and Poustka 2006) was used, which has demonstrated satisfactory internal consistency (α = .83) and convergent validity (Bölte et al. 2008).

### Data Analyses

The study data were analyzed using the Statistical Package for the Social Sciences (SPSS) version 25, Mplus version 7.4 (Muthén and Muthén 1998–2012), and Microsoft Excel.

**Fig. 1:**
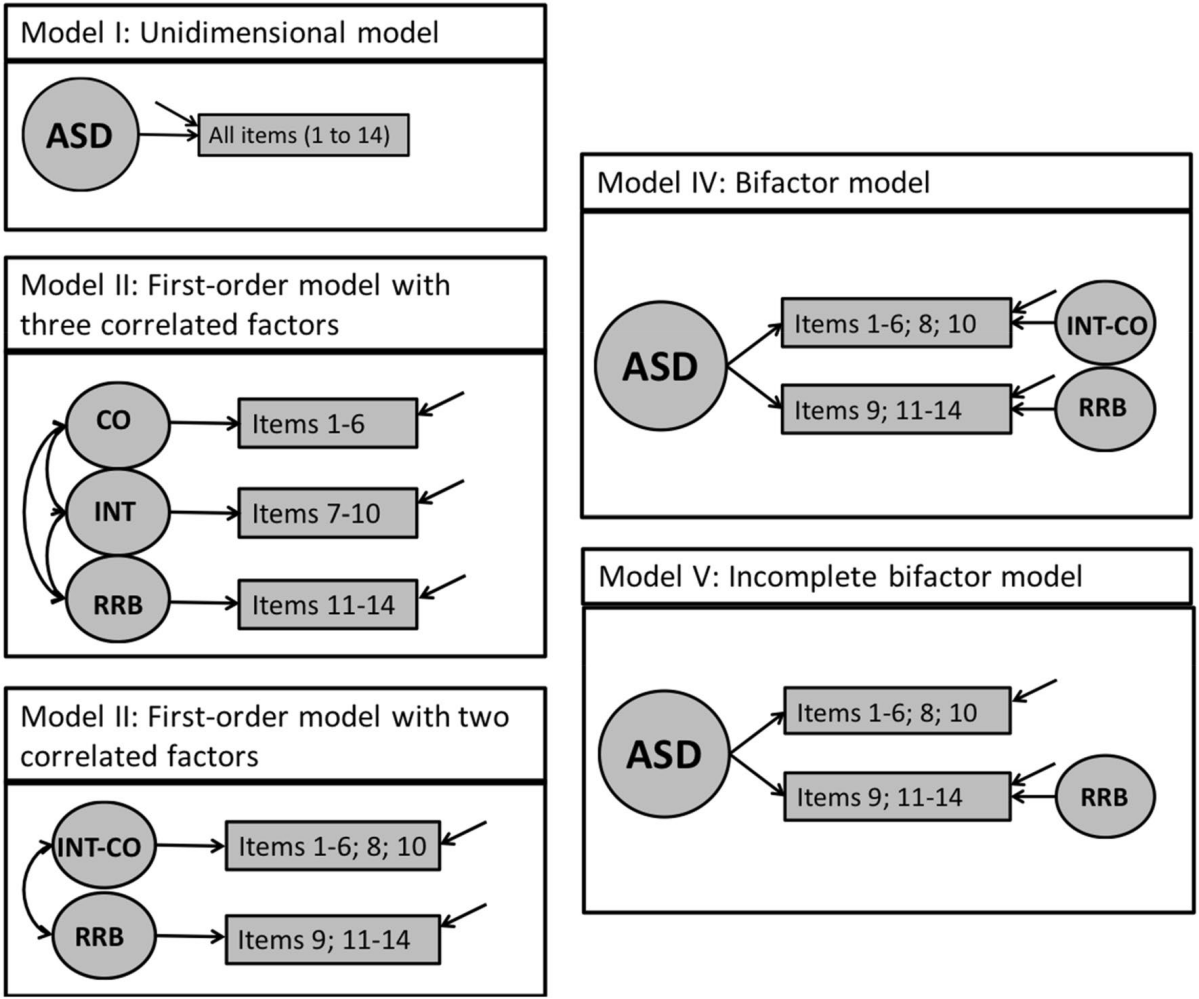
Possible alternative factor structures underlying the Symptom Checklist for Autism Spectrum Disorders (SCL-ASD) which were examined by the use of confirmatory factor analysis. *ASD* autism spectrum disorders, *INT* social interaction, *CO* communication and language, *INT-CO* interaction and communication, *RRB* restricted, repetitive behaviors

First, confirmatory factor analyses (CFA) were performed using Mplus to examine the factor structure of the SCL-ASD. Here, due to the ordinal structure of our data (4-point Likert-type scale), the robust weighted least squares with mean and variance adjustment estimator (WLSMV) was used for model estimation (Brown 2006; Muthén and Muthén 1998–2012). To handle missing data, the default procedure for WLSMV in Mplus was employed (pairwise present analysis; Muthén and Muthén 1998–2012). Overall, five different models specified a priori were tested and compared (see Fig. 1). First, a one-factor model, suggesting one general factor of autism that influences all items, was examined to support the scoring and interpretation of the total score (unidimensional model; model I). Second, a model with three correlated factors consistent with the DSM-IV and ICD-10 structure was tested (factor 1: CO, factor 2: INT, factor 3: RRB; model II). Third, a model with two correlated factors according to the DSM-5 and ICD-11 diagnostic criteria was specified (factor 1: INT-CO, factor 2: RRB; model III). Models II and III both imply that each factor influences a subset of items. Fourth, a bifactor model was examined (model IV) to check the existence of a general ASD factor that accounts for variance in item scores and the existence of additional specific factors (factor 1: INT-CO, factor 2: RRB) which further explain variance in item subsets that cannot be attributed to the general factor (Chen et al. 2013). Due to the results for the bifactor model (see results section), we finally evaluated an incomplete bifactor model with one general ASD factor and one specific factor (RRB; model V).

Model fit was assessed using several fit indices. First, the Chi-square (χ^2^) fit statistic and the χ^2^/df ratio were considered. If the p value associated with the χ^2^ value is greater than .05, the test indicates that the examined model fits the data. As the χ^2^ value tends to become significant in large samples even in the case of actually acceptable models, Schermelleh-Engel et al. (2003) recommend using the ratio χ^2^/df. This ratio should be as small as possible to indicate a good model fit. A ratio of “3” represents an “acceptable” data-model fit and a ratio of “2” a “good” data-model fit (Schermelleh-Engel et al. 2003). Furthermore, we relied on the root mean square error of approximation (RMSEA), the comparative fit index (CFI), the Tucker-Lewis Index (TLI) and the Standardized Root Mean Square Residual (SRMR) to evaluate model fit. According to Browne and Cudeck (1993), RMSEA values ≤ .05 indicate good and values between .05 and .08 indicate adequate fit. Regarding the CFI and the TLI, values ≥ .95 can be considered as good and values ≥ .90 can be considered as acceptable (Schreiber et al. 2006). The SRMR should be less than .05 for a good fit (Hu and Bentler 1995), whereas values smaller than .10 may be interpreted as acceptable.

Moreover, nested models were compared using the Chi-square difference test available in Mplus (Muthén and Muthén 1998–2005). The correlated-factors model with two factors (model III) is nested within the less restricted bifactor model with two specific factors and the incomplete bifactor model with one specific factor. The incomplete bifactor model is nested within the less restricted complete bifactor model. If the Chi-square difference test is significant, the less restricted model should be retained, and if it is nonsignificant, the more restricted model should be favored (Schermelleh-Engel et al. 2003). Moreover, we computed differences in CFI, RMSEA and SRMR to compare nested models (cf. Cheung and Rensvold 2002). For the interpretation of these differences, see the respective information regarding measurement invariance below. Furthermore, the factor structure and factor loadings were examined. Following Kline (1994), factor loadings of > 0.30 were considered as acceptable.

Due to the large age-range in our study, we additionally tested for measurement invariance to examine whether the same construct was assessed across different age groups. As a result of the relatively small sample size in relation to the large number of freely estimated parameters in measurement invariance analyses, we were only able to divide the sample into two age groups, the first one ranging from 2 to 10 years (*n* = 155) and the second one ranging from 11 to 18 years of age (*n* = 157). We performed the sample division based on a median split in order to obtain groups of similar size. The first level of measurement invariance—configural invariance—requires the same items to load onto the same latent factors in each group. For the next level—metric invariance—it must also be ensured that the loading of each item on the latent factor is equal across groups. The third level—scalar invariance—further demands item thresholds to be equal across groups. Since the chi-square statistic is sensitive to sample size, it has been recommended to compute goodness of fit indexes to evaluate measurement invariance (for a detailed overview see Chen 2007). The same fit indices with the same cut-off values as mentioned above are valid here. Additionally, for metric invariance, a decline larger than − .010 in CFI between the two levels of invariance tests combined with a change of ≥ .015 in RMSEA or a change of ≥ .030 in SRMR would indicate noninvariance; for scalar invariance, a decline larger than − .010 in CFI together with a change of ≥ .015 in RMSEA or a change of > .010 in SRMR would indicate noninvariance (Cheung and Rensvold 2002; Chen 2007).

The reliability of the SCL-ASD scale scores was examined using ordinal alpha (Zumbo et al. 2007). As alpha is not recommended for estimating the reliability of bifactor models, omega statistics (omega ω; omega hierarchical ω_H_; omega hierarchical subscale ω_S_) were additionally calculated (Brunner et al. 2012). Omega (ω) displays the amount of variance in item subsets or all items accounted for by the general factor and the specific factors taken together. Omega hierarchical (ω_H_) reflects the amount of variance in item subsets or the complete item pool explained by the general factor, while omega hierarchical subscale (ω_S_) expresses the amount of variance attributable to a specific factor (Brunner et al. 2012).

Finally, to assess the convergent and divergent validity of the SCL-ASD, Pearson’s correlation coefficients (*r*) between the SCL-ASD total score and subscale scores and between the total score and the subscale scores of the MBAS, the ADI-R, the CBCL and the SCQ were considered. As there was a wide variation in age in our sample and as autistic traits may present quite differently at different ages, we introduced age as a covariate in all correlation analyses. To test the difference between correlations for significance, we used the Hittner et al. (2003) method.

## Results

### Confirmatory Factor Analyses

Table 2 summarizes the results of the CFAs. In terms of the χ^2^/*df* ratio, the CFI and the TLI, the correlated-factors model with two factors (model III) and the bifactor model with two specific factors (model IV) provided a good fit to the data. Furthermore, the SRMR value indicated a good fit for the bifactor model (model IV) and an acceptable fit for the correlated-factors model with two factors (model III); the RMSEA value indicated an adequate fit for both models.

**Table 2:** Confirmatory factor analyses comparing alternative models of the SCL-ASD (Estimator: WLSMV)

Model	χ^2^	*df*	χ^2^/*df*	*p*	CFI/TLI	RMSEA (CI)	SRMR	Δχ^2^	*df*	ΔCFI	ΔRMSEA	ΔSRMR
I. Unidimensional model	361.09	77	4.69	< .01	.905/.888	0.109 (0.098–0.120)	.089	–	–			
II. First-order correlated-factors model (three factors)	245.78	74	3.32	< .01	.943/.929	0.086 (0.074–0.098)	.071	–	–			
III. First-order correlated-factors model (two factors)	183.56	64	2.87	< .01	.959/.950	0.077 (0.064–0.091)	.066	–	–			
IV. Bifactor model (two specific factors: INT-CO and RRB)	121.63	52	2.33	< .01	.976/.964	0.066 (0.050–0.081)	.048	–	–			
V. Incomplete bifactor model (one specific factor: RRB)	175.141	60	2.92	< .011	.961/.946	0.078 (0.065–0.092)	.061	–	–			
III vs. IV	–	–	–	–	–	–	–	58.657*	12	–.017	.011	.018
III vs. V	–	–	–	–	–	–	–	12.853*	4	–.002	− .001	.005
V vs. IV	–	–	–	–	–	–	–	49.592*	8	–.015	.012	.013

Sample size: *n* = 312

*SCL-ASD* Symptom Checklist for Autism Spectrum Disorders, *WLSMV* robust weighted least squares with mean and variance adjustment estimator, *χ*^*2*^ empirical χ^2^ value, *df* degrees of freedom, *p* empirical significance value, *CFI* comparative fit index, *CI* confidence interval, *TLI* Tucker–Lewis Index, *RMSEA* root mean square error of approximation, *SRMR* standardized Root Mean Square Residual, *Δχ*^*2*^ corrected difference between χ^2^ values of two competing models for difference testing,

*Δχ^2^ test significant at the level of 1%

In contrast, the unidimensional model (model I) and the correlated-factors model with three factors (model II) showed a worse fit. The CFI/TLI and the SRMR values were in an acceptable range, but the other fit indices did not meet the cut-off criteria. Model I and model II are therefore not considered in the further analyses.

Comparing models III and IV, the bifactor model with two factors (model IV) showed similar, but slightly better fit indices. The result of the Chi-square difference test and the difference in CFI between the two models also indicated that model IV should be preferred over model III. However, this finding was not supported by the difference in RMSEA between the two models, which slightly missed the cut-off for non-equivalence. In line with this, the correlation between the two dimensions in model III was high (*r* = .71), which suggests an underlying general factor (as proposed by model IV).

With regard to the standardized parameter estimates (see Table 3), in the bifactor model (model IV), all items loaded significantly on the general factor and exceeded the minimum threshold of λ = .30. In addition, except for item 11, all items of the *RRB* subscale showed substantial loadings on the specific RRB factor. Yet, most of the items of the *INT-CO* subscale showed weak, partially non-significant or even negative loadings on their specific factor. This calls into question the factorial validity of the *INT-CO* subscale within the bifactor model.

**Table 3:** Standardized factor loadings and standard errors (in brackets) of the first-order correlated-factors model with two factors (III), the bifactor model (IV) and the incomplete bifactor model (V) of the SCL-ASD in the total sample

Item	Description	Model III		Model IV			Model V	
		INT-CO	RRB	Total scale	INT-CO	RRB	Total scale	RRB
1	Marked impairment in the use of multiple nonverbal behaviors such as eye to-eye gaze, facial expression, body postures, and gestures to regulate social interaction	0.70 (0.04)		0.68 (0.04)	0.21 (0.08)		0.69 (0.04)	
2	Failure to develop peer relationships appropriate to developmental level	0.72 (0.04)		0.71 (0.04)	0.14 (0.09)^ns^		0.72 (0.04)	
3	Lack of emotional reciprocity/empathy; no emotional response to the emotions of others	0.79 (0.30)		0.79 (0.03)	-0.03 (0.09)^ns^		0.79 (0.03)	
4	Lack of social adaptation skills	0.58 (0.05)		0.62 (0.06)	-0.46 (0.11)		0.58 (0.05)	
5	Incongruent affective communication—expressions, behavior and emotions do not match up	0.75 (0.04)		0.77 (0.04)	-0.20 (0.09)		0.75 (0.04)	
6	A lack of spontaneous seeking to share enjoyment, interests, or achievements with other people	0.77 (0.03)		0.76 (0.04)	0.19 (0.09)		0.77 (0.03)	
8	In individuals with adequate speech, marked impairment in the ability to initiate or sustain a conversation with others	0.65 (0.04)		0.62 (0.06)	0.52 (0.10)		0.65 (0.04)	
9	Lack of varied, spontaneous make-believe play or social imitative play appropriate to developmental level	0.71 (0.04)		0.71 (0.04)	0.12 (0.07)^ns^		0.71 (0.04)	
10	Stereotyped and repetitive use of language or idiosyncratic language		0.76 (0.04)	0.54 (0.05)		0.54 (0.06)	0.54 (0.05)	0.55 (0.06)
11	Encompassing preoccupation with one or more stereotyped patterns of interest that is abnormal either in intensity or focus		0.55 (0.05)	0.45 (0.05)		0.23 (0.07)	0.45 (0.05)	0.24 (0.07)
12	Apparently inflexible adherence to specific, nonfunctional routines or rituals		0.87 (0.03)	0.64 (0.04)		0.54 (0.06)	0.63 (0.04)	0.54 (0.06)
13	Stereotyped and repetitive motor mannerisms (e.g., hand or finger flapping or twisting, or complex whole-body movements)		0.68 (0.04)	0.43 (0.05)		0.65 (0.06)	0.42 (0.05)	0.66 (0.06)
14	Persistent preoccupation with parts of objects		0.70 (0.04)	0.49 (0.05)		0.51 (0.07)	0.49 (0.05)	0.51 (0.06)

Sample size *n* = 312

*SCL-ASD* Symptom Checklist for Autism Spectrum Disorders, *INT-CO* interaction and communication, *RRB* restricted, repetitive behaviors, *ns* nonsignificant loading

Hence, we constructed an incomplete bifactor model, excluding the specific INT-CO factor (model V). As can be seen in Table 2, model V showed a satisfactory model fit based on conventional criteria (χ^2^/*df*, CFI/TLI ‘good’; RMSEA ‘adequate’; SRMR ‘acceptable’). However, the fit indices were slightly worse than those for model IV, and the result of the Chi-square difference test as well as the difference in CFI between the two models also indicate that the complete bifactor model (model IV) should be preferred over the incomplete model (model V; see Table 2). However, the difference in RMSEA was just below the cut-off for non-equivalence. Moreover, model V had the advantage that it did not yield any weak, non-significant or even negative loadings (see Table 3).

Additional analyses for the bifactor model (model IV) showed that we can assume measurement invariance between the two age groups on a configural, metric and scalar level. CFI and TLI are above .95 on all levels and can accordingly be considered as good. The RMSEA and the SRMR are in an adequate range on all levels. Furthermore, the changes in CFI, RMSEA and SRMR indicate metric and scalar invariance (see Table 4).

**Table 4:** Results of measurement invariance tests of the bifactor-model across age groups

Level of measurement invariance	*df*	CFI	TLI	RMSEA (90% CI)	SRMR	∆CFI	∆RMSEA	∆SRMR
Configural invariance	104	.972	.958	.071 (.054; .087)	.049			
Metric invariance	127	.977	.972	.058 (.041; .074)	.060	.005	− .013	.011
Scalar invariance	163	.975	.976	.054 (.038; .068)	.062	− .002	− .004	.002

The sample was divided into two groups based on a median split: 2–10 years (*n* = 155)/11–18 years (*n* = 157)

*df* degrees of freedom, *CFI* comparative fit index, *TLI* Tucker–Lewis Index, *RMSEA* root mean square error of approximation, *CI* Confidence interval, *SRMR* Standardized Root Mean Square Residual, *∆* difference

### Reliability

The total scale and the two subscales of the modified SCL-ASD demonstrated good internal consistency (see Table 5). Ordinal alpha exceeded .70 for all scales; item-subscale correlations were mostly moderate to high (*r*_it_ = .38–.70). With regard to the bifactor model with two factors (model IV), the amount of variance attributable to the total scale and the subscales taken together, as displayed by omega, was .93 for the total scale, .90 for the *INT-CO* scale and .86 for the *RRB* scale. When considering all items, the general scale explained most of the variance (ω_H_ = .85). Regarding the items of the *RRB* scale, both the general factor and the specific RRB factor accounted for a substantial amount of variance (see Table 5). However, by far the most variance in the item subset belonging to the *INT-CO* subscale was accounted for by the general factor, while ω_S_ proved to be very low (.01; see Table 5). Considering the incomplete bifactor model (model V), ω was .92 for the total scale and .84 for the *RRB* domain. With regard to all items, again, most of the variance was attributable to the general scale (ω_H_ = .84); regarding the items of the *RRB* domain, both the general factor and the specific factor accounted for item variance (ω_H_ = .43, ω_S_ = .42).

**Table 5:** Descriptive statistics, internal consistencies, part-whole corrected item-scale correlations, range of factor loadings and omega statistics of the bifactor model of the SCL-ASD

Variable	Number of items	*M*	*SD*	α	Range of *r*_it_	Range of factor loadings	ω	ω_H_	ω_S_
Total score	13	1.23	0.67	0.90	0.43–0.66	0.43–0.77	0.93	0.85	–
Communication/interaction (INT-CO)	8	1.32	0.71	0.88	0.44–0.70	0.12–0.46	0.90	0.89	0.01
Restricted, repetitive behaviors (RRB)	5	1.07	0.79	0.78	0.38–0.67	0.23–0.65	0.86	0.47	0.39

Sample size *n* = 312

*SCL-ASD* Symptom Checklist for Autism Spectrum Disorders, *M* mean (items rated on a 4-point Likert scale ranging from 0 to 3), *SD* standard deviation, *α* Ordinal alpha, *r*_*it*_ part-whole corrected item-scale correlations, *ω* omega (amount of variance accounted for by the total scale and the subscales taken together), *ω*_*H*_ omega hierarchical (amount of variance accounted for by the total scale), *ω*_*S*_ omega hierarchical subscale (amount of variance accounted for by the subscale)

### Validity

The correlations between the SCL-ASD total score and subscale scores and the MBAS, ADI-R, CBCL and SCQ are displayed in Table 6. All correlations are adjusted for age. Since the SCL-ASD *INT-CO* and *RRB* subscales correlate strongly, partial correlations were calculated in a further step: Correlations between the *INT-CO* subscale and the MBAS, ADI-R, CBCL and the SCQ were adjusted for the influence of the SCL-ASD *RRB* subscale. Correlations between the *RRB* subscale and the MBAS, ADI-R, CBCL and SCQ were adjusted for the influence of the *INT-CO* subscale.

**Table 6:** Correlations and partial correlations (in brackets) between the SCL-ASD (DSM-5/ICD-11) and the MBAS, the ADI-R, the CBCL and the SCQ (all correlations are adjusted for age)

	Modified SCL-ASD (DSM-5/ICD-11)			
	*n*	Total score	Interaction/communication (INT-CO)	Restricted, repetitive behavior (RRB)
MBAS	142			
Total score		0.71	0.69 (0.54)	0.56 (0.29)
Theory of mind, social contact and play		0.55	0.58 (0.49)	0.37 (0.05)
Joint attention, facial expression, gesture		0.64	0.66 (0.55)	0.44 (0.11)
Stereotyped and inadequate behavior		0.71	0.60 (0.34)	0.68 (0.51)
Special interests, conspicuous speech, motor activity		0.31	0.27 (0.15)	0.29 (0.17)
ADI-R	193–195			
Social interaction (INT)		0.59	0.58 (0.42)	0.46 (0.16)
Communication and language (CO)		0.52	0.50 (0.32)	0.44 (0.19)
Restricted/repetitive behaviors (RRB)		0.46	0.35 (0.03)	0.53 (0.42)
SCQ	170			
Total score		0.68	0.62 (0.41)	0.60 (0.35)
CBCL	144–149			
Total score		0.35	0.37 (0.30)	0.23 (0.03)
Externalizing		0.11	0.16 (0.20)	-0.01 (-0.14)
Internalizing		0.31	0.30 (0.21)	0.24 (0.10)
Aggressive behavior		0.11	0.16 (0.18)	0.01 (-0.10)
Anxious/depressed		0.21	0.19 (0.11)	0.18 (0.10)
Attention problems		0.36	0.36 (0.25)	0.28 (0.10)
Rule-breaking behavior		0.10	0.19 (0.27)	-0.05 (-0.20)
Somatic complaints		0.12	0.10 (0.03)	0.12 (0.09)
Social problems		0.22	0.27 (0.26)	0.09 (-0.07)
Thought problems		0.45	0.36 (0.15)	0.45 (0.32)
Withdrawn		0.36	0.36 (0.27)	0.27 (0.09)

*SCL-ASD* Symptom-Checklist for Autism Spectrum Disorders, *MBAS* Marburg Rating Scale for Asperger's Syndrome, *ADI* Autism Diagnostic Interview-Revised, *SCQ* Social Communication Questionnaire, *CBCL* Child Behavior Checklist

Predominantly, moderate to high correlations were found between the SCL-ASD and the MBAS subscales and total scales. Exceptions were the lower correlations of both the SCL-ASD *INT-CO* and *RRB* subscale with the MBAS subscale S*pecial Interests, Conspicuous Speech, and Motor Activity*. The correlations of the MBAS subscales *Theory of Mind, Social Contact and Play* and *Joint Attention, Facial Expression, Gesture* with the SCL-ASD *INT-CO* subscale were significantly higher than the correlations of these two MBAS subscales with the SCL-ASD *RRB* subscale (*Z* = 3.27, *p* = .001; *Z* = 3.67, *p* < .001). Interestingly, the correlations of the SCL-ASD *RRB* subscale with most of the MBAS scales were substantially reduced when controlling for the influence of the *INT-CO* items. Here, only the correlation with the MBAS subscale on S*tereotyped and Inadequate Behaviour*, which captures a similar construct as the *RRB* scale, remained comparatively high. On the other hand, the moderate correlation of the SCL-ASD *INT-CO* subscale with the MBAS scale on *Stereotyped and Inadequate Behaviour* was substantially reduced when controlling for the influence of the *RRB* subscale.

Moderate to high correlations also emerged between the SCL-ASD subscales and total scale and the ADI-R subscales. With respect to the *INT-CO* subscale of the SCL-ASD, the correlations with the ADI-R *INT* subscale and the ADI-R *CO* subscale were significantly higher than the correlation with the ADI-R *RRB* subscale (*Z* = 3.90, *p* < .001 and *Z* = 2.40, *p* = .02, respectively). Additionally, they were more stable when controlling for the influence of the SCL-ASD *RRB* subscale; the correlation between the SCL-ASD *INT-CO* subscale and the ADI-R *RRB* subscale dropped to almost zero when controlling for the SCL-ASD *RRB* subscale. For the *RRB* subscale of the SCL-ASD, the highest associations were found with the ADI-R *RRB* subscale, even when controlling for the SCL-ASD *INT-CO* subscale. However, this correlation was not significantly higher than the correlation between the SCL-ASD *RRB* subscale and the ADI-R *INT* subscale (*Z* = 1.20, *p* = 0.23) and the ADI-R *CO* subscale *(Z* = 1.51*, p* = 0.13*)*.

Comparatively high correlations emerged between the SCQ total scale and the SCL-ASD total scale and subscales. There was no significant difference between the correlation of the SCQ with the SCL-ASD *RRB* subscale and the correlation of the SCQ with the SCL-ASD *INT-CO* subscale *(Z* = − 0.39*, p* = 0.70*)*. The correlations on the subscale level remained moderate when the influence of the other subscale was controlled for.

Low to moderate correlations were detected between the SCL-ASD scales and the CBCL subscales and total scales. With few exceptions, the correlations of the SCL-ASD scales with the CBCL syndrome scales were lower than the correlations between the SCL-ASD subscales and the subscales of the other measures of autistic traits. More precisely, the correlation between the SCL-ASD total score and the CBCL total score was significantly weaker than the correlation between the SCL-ASD and MBAS total scores (*Z* = − 5.09, *p* < .001) and the correlation between the SCL-ASD and SCQ total scores (*Z* = − 4.21, *p* < .001). Also, the correlation between the SCL-ASD total score and the CBCL total score was significantly weaker than the correlation between the SCL-ASD total score and the ADI-R *INT* subscale (*Z* = − 2.84, *p* = .005). The differences between the correlation of the SCL-ASD total scale with the CBCL total scale and the correlations of the SCL-ASD total scale with the ADI-R *CO* and *RRB* subscales were not significant (*Z* = − 1.93, *p* = 6; *Z* = − 1.13, *p* = .26).

## Discussion

This study examined the structure of DSM- and ICD-defined ASD symptoms as well as the psychometric properties of the German SCL-ASD in a sample of clinically referred children and adolescents aged 2 to 18 years. Regarding the fit indices, the results of confirmatory factor analyses most likely support the presence of a bifactor model with a general ASD factor and two specific group factors, INT-CO and RRB. Measurement invariance analyses on a configural, metric and scalar level suggest that with the bifactor model the same construct is assessed in both age groups. This finding is in line with the DSM-5 and ICD-11 symptom domains and provides support for the decision to consider two symptom domains instead of three, as was the case in former versions of the classification systems. All SCL-ASD items loaded significantly on the general ASD factor, indicating that all DSM- and ICD-defined items belong to a common concept.

The item loadings on the specific RRB factor were also significant and substantially large, while the items of the INT-CO subdomain demonstrated weak, partially non-significant or negative loadings on their specific factor. This questions the factorial validity of the specific INT-CO factor within the bifactor model and limits the interpretation of our results.

The results of our analyses differ from previous results in several aspects. Actually, some studies yielded support for a strong general ASD factor (Murray et al. 2017) or a bifactor model of ASD traits with two domain-specific factors (Posserud et al. 2013). However, in the only other study (to our knowledge) to find a satisfactory fit of a bifactor model with two domain-specific factors, some items showed only low loadings on the general factor and, thus, the general factor was quite weak (Posserud et al. 2013). In our study, by contrast, the general ASD factor proved to be quite strong, while the specific INT-CO factor was only^2012^ weakly defined. Moreover, several previous studies yielded satisfactory results for correlated-factors models including different numbers of factors (e.g., Beuker et al. 2012; Frazier et al. 2014; Guthrie et al. 2013; Sipes and Matson 2014), and some studies examining bifactor models of ASD traits were unable to establish superiority over correlated-factors models (Lecavalier et al. 2009; Snow et al. 2009).

As indicated by the omega statistics, the general ASD factor accounted for a high proportion of variance in the item scores in the complete bifactor model, again highlighting the strength of this common factor. Moreover, the specific RRB factor explained additional variance in item scores. On the other hand, barely any variance was attributable to the specific INT-CO factor. Although the construction of an incomplete bifactor model excluding the specific INT-CO factor eliminated the problems of low, non-significant or negative item loadings in the model and the low amount of variance accounted for by the specific INT-CO factor, the bifactor model with two specific symptom domains provided a better fit to the data than this alternative model. Of note, however, if a priori specified and embedded in a theoretical context, a bifactor model excluding one specific factor might be psychometrically sounder and allow for a clearer interpretation than a bifactor model with weakly defined specific factors (as for example indicated by non-significant or negative item loadings; cf. Eid et al. 2017; Junghänel et al.^2020^). In such an a priori defined model, the items of the domain which is not modeled as specific factor mainly define the meaning of the general factor (Eid et al. 2017). As the incomplete bifactor model yielded a satisfactory data fit in our analyses and has the potential to overcome some problems with the complete bifactor model, it might be worth considering this model in future research despite its slightly worse fit compared to the complete bifactor model.

To our knowledge, no previous study has yielded a comparably weak INT-CO factor. However, many previous studies regarded correlated-factors models without testing for the existence of an additional general factor, which might weaken the contribution of special subscales to explained variance. Thus, the results of our study require replication in future studies. In light of our findings, when interpreting data for the SCL-ASD *INT-CO* subscale, clinicians and researchers should keep in mind that the items of this scale are mainly influenced by the general ASD factor and barely reflect an independent construct.

The present study is, to our knowledge, the first study analyzing the measurement invariance of a bifactor model of ASD, which is consistent with DSM-5 criteria, across age groups. Therefore, it is difficult to compare the results to previous studies. However, of note, Duku et al. (2013) were not able to establish measurement invariance for a second-order model consistent with DSM-5 criteria. Instead, they found a first-order six-factor model to provide good fit and to be invariant across several subgroups (divided by age, sex, verbal ability). Given these results and some shortcomings of our current analyses (i.e., small sample size, uneven age distribution; see below), the question of which model is most suitable in terms of data fit and measurement invariance across age groups remains to be examined further.

Internal consistencies were satisfactory for both the SCL-ASD subscales and the total scale, and most of the part-whole corrected item-(sub)scale correlations were moderate to high, supporting the reliability of the scales and items. Notably, the SCL-ASD *INT-CO* subscale demonstrated satisfactory internal consistency, while ω_S_ (as a reliability estimate based on the bifactor model) was close to zero. This finding may be explained by the strong general ASD factor. Ordinal alpha does not distinguish between the influence of a general construct underlying all items and the influence of a specific subscale, which might both contribute to the high internal consistency of a subscale (cf. Reise et al. 2007). The omega statistics, on the other hand, allow for a differentiation between the amounts of variance accounted for by the general ASD factor versus the specific subscales (Reise et al. 2007).

In line with our hypotheses, the SCL-ASD subscales and total scale generally showed mainly significantly higher correlations with other measures of ASD traits than with measures of other externalizing and internalizing symptoms, thus hinting at the convergent and divergent validity of the questionnaire. In particular, moderate to high correlations were found between the SCL-ASD subscales and subscales of other instruments assessing similar constructs, e.g. between the SCL-ASD *RRB* subscale and the subscale on *Stereotyped and Inadequate Behaviour* of the MBAS and the *RRB* subscale of the ADI-R or between the SCL-ASD *INT-CO* subscale and the MBAS subscales on *Theory of Mind, Social Contact and Play* and *Joint Attention, Facial Expression and Gesture* and the ADI-R *INT* subscale. However, even these correlations did not show perfect correspondence of the constructs. Regarding the correlations of the ADI-R subscales with the SCL-ASD subscales, this might also be partly due to the different raters of these instruments.

Some limitations of the present study should be mentioned: First, the wide variation in the ages of the children included in the sample is a disadvantage of the study. To account for this shortcoming, we additionally tested for measurement invariance. However, as a result of the relatively small sample size in relation to the large number of freely estimated parameters in measurement invariance analyses and the age distribution in our sample, we were only able to divide the sample into two age-groups. Thus, our analyses might be biased and require replication in larger samples with a more even age distribution. Furthermore, for the calculation of the convergent and divergent validity, we included age as a covariate. Second, a methodological limitation of the study is that we did not calculate the internal reliability of all scales used in this study in our current sample. This was due to the method of data collection. Data were collected during routine clinical care; patients complete the SCL-ASD and the other measures used in this study as part of the intake assessment. Data are steadily entered in a database on the scale level. Thus, some data were not available to us at the item level. However, various studies have shown the internal consistencies of these scales in other samples (see measures section). Third, based on previous studies on the structure of autistic traits and the grouping of symptoms in the ICD-10 and DSM-IV (which formed the basis for the development of the SCL-ASD), we derived several hypotheses about the SCL-ASD factor structure and examined the fit of these structures using CFA. Another, also appropriate possibility would have been to conduct an exploratory factor analysis (EFA) so as to allow for more flexibility and to make sure that no meaningful solutions were overlooked. In the field of bifactor models, the Schmid-Leiman and Jennrich Bentler exploratory bifactor approach (Mansolf and Reise 2016) is promising to analyze the items’ higher relations. Unfortunately, the sample size of this study was too small to conduct both an EFA and a CFA. However, the conduction of exploratory bifactor analyses might be an interesting direction for future research. Fourth, the loadings of the items on the specific INT-CO factor in the bifactor model were very heterogeneous, rendering it difficult to interpret them and to consider them as belonging to a common construct. In addition, we chose Kline’s (1994) criterion for the interpretation of factor loadings, considering loadings ≥ .30 as satisfactory. Other authors, like Matsunaga (2010), consider values equal or greater than .40 as acceptable. Fifth, as we only considered the factor structure of parent-rated ASD symptoms, the results need to be replicated in samples including self-report and clinician-rated data. Finally, the discriminant validity of the SCL-ASD between children and adolescents with and without ASD remains to be examined.

## Conclusion

To conclude, the SCL-ASD is a reliable and valid instrument to assess ASD traits. The results of our confirmatory factor analyses most likely support a bifactor model of ASD traits with a general ASD factor and two domain-specific factors (INT-CO and RRB), whereby only the RRB factor accounts for a substantial amount of item variance when controlling for the influence of the general factor. Although limited by the lacking contribution of the specific INT-CO factor to explain item variance, this structure is generally consistent with the DSM-5 and ICD-10 symptom domains. As studies examining the factor structure of ASD symptoms have yielded equivocal results, more research is required to further illuminate the underlying structure of ASD symptoms. Besides a further consideration of bifactor models, approaches integrating previous findings (Kim et al. 2018) might be of special interest.

## Supplementary information

Below is the link to the electronic supplementary material.

Supplementary material 1 (DOCX 19 kb)
