# Supplementary material for: Dimensions of Autistic Traits Rated by Parents of Children and Adolescents with Suspected Autism Spectrum Disorders
Source: J Autism Dev Disord. 2021 Jan 8;51(11):3989–4002. doi: 10.1007/s10803-020-04850-4 (PMC8510981; doi:10.1007/s10803-020-04850-4)
Supplement: Supplementary file 1 — Supplementary material 1 (DOCX 19 kb) [file 10803_2020_4850_MOESM1_ESM.docx]

Table S1.

*Overview of studies that addressed the question of the existence of a general factor versus specific factors of autism.*

|  | Snow et al. (2009) | Posserud et al. (2013) | Murray et al. (2017) |
| --- | --- | --- | --- |
| Sample | Individuals with pervasive developmental disorders (PDD) | General population of adolescents (fourth wave of the longitudinal Bergen Child Study) | Individuals with and without a clinical diagnosis of autism spectrum disorder |
| Sample size *n* | 1861 | 10220 | 562 (*n* = 147 individuals with ASD) |
| Gender | 1455 males, 406 females | not reported | 204 males, 357 females, 1 not reported |
| Age ranges | 4 to 18 years (*M* = 8.3; *SD* = 3.2) | 16 to 19 years | 18 to 69 years (*M* = 30.6; *SD* = 11.8) |
| Outcome measure | The Autism Diagnostic Interview-Revised (ADI-R) | The Autism Symptom Self-Report for adolescents and adults (ASSERT) | Autism Spectrum Quotient (AQ); Autism Spectrum Quotient Short Form (AQ-S) |
| Statistical procedure | EFA/CFA | EFA/CFA | CFA |
| Major results | EFA  The two-factor solution was the most interpretable one. It showed a pattern of factor loadings that indicated a distinction between social/communication items and restricted/repetitive behavior items.  CFA  Based on fit indices, the two-factor model of social/ communication items and restricted/repetitive behaviors provided similar fit as the three-factor solution and was superior to the one-factor solution. Models based on a bifactor structure did not yield better fit indices. | EFA  The two- and three-factor EFA solutions showed good to excellent fit while the one-factor model was definitely discarded.  CFA  The best statistical model fit was found for a bifactor model with one general factor and two domain-specific factors tied to social difficulties on the one hand and rigid and repetitive behavior and interests on the other hand. | Model fit for a bifactor model was good according to all model fit indices and better than that of a first-order correlated-factors model. The results supported the inclusion of the general factor. |

*Note.* CFA = confirmatory factor analysis, EFA = exploratory factor analysis, *M* = mean, *SD* = standard deviation.
